# Application of the Multi-Omics Liquid Biopsy Method M2P-HCC in Early Liver Cancer Screening for High-Risk Individuals with Hepatitis B-Related Liver Cancer

**DOI:** 10.3390/diagnostics13152484

**Published:** 2023-07-26

**Authors:** Xian Yu, Xuezhong Lei

**Affiliations:** Center of Infectious Diseases, West China Hospital of Sichuan University, Chengdu 610041, China; yuxian2712@163.com

**Keywords:** hepatocellular carcinoma, liquid biopsy, HBV, ctDNA, early diagnosis

## Abstract

Background: Hepatocellular carcinoma (HCC) is one of the most common malignant tumors worldwide, with low rates of early diagnosis and surgical resection. In recent years, with the rapid development of liquid biopsy technology, circulating tumor DNA (ctDNA) has emerged as a research hotspot in the field of precision medicine for liver cancer. Existing studies have demonstrated the suitability of ctDNA for combined detection with other liver cancer diagnostic markers, enabling a multi-index analysis. In recent years, a novel prediction model has been developed for early liver cancer screening based on ctDNA liquid biopsy, M2P-HCC (methylation, mutation, and protein-HCC), mainly incorporating methylation changes, gene mutations, and protein markers associated with liver cancer. Preliminary validation in the HCCscreenTM Investigational (HIT, ChiCTR1800020233) study, which focused on screening early liver cancer in communities with Hepatitis B surface antigen (HBsAg) positivity, yielded promising results with 100% sensitivity and 94% specificity. However, it remains uncertain whether M2P-HCC can be effectively applied in high-risk populations for Hepatitis B-associated liver cancer, warranting further research. Methods: Patients who were under long-term follow-up at the outpatient clinic of the Infectious Diseases Center of West China Hospital of Sichuan University from December 2020 to January 2023 were recruited in this prospective observational study and underwent the M2P-HCC test. The study population consisted of high-risk patients with Hepatitis B-related liver cancer who met the inclusion criteria. Patients with a history of previous malignancy, recent blood transfusion, autoimmune diseases, and human immunodeficiency virus (HIV) infection were excluded. Clinical data were collected at a baseline, and all patients underwent the M2P-HCC blood test. Based on the test results, they were categorized into positive, early-warning, and negative groups. Prospective cohort observation and regular follow-ups were performed for 6–8 months. Results: 313 patients met the inclusion criteria and were included in the study. After 6–8 months of follow-up, HCC occurred in 41(13.1%) participants. The M2P-HCC test demonstrated good predictive performance with an area under the curve (AUC) of 0.88 (95% CI: 0.81–0.95, *p* < 0.001) and a cutoff value of 83 points (sensitivity 82.9% and specificity 85.7%). In contrast, the combination of alpha-fetoprotein (AFP) and ultrasound (US) yielded an inferior predictive performance (AUC 0.76 (95% CI: 0.69–0.84, *p* < 0.001), sensitivity 58.5%, and specificity 94.1%). Multivariate analyses revealed that M2P-HCC was an independent predictor of increased risk of HCC (OR = 1.16 [1.09–1.22], *p* < 0.001). Conclusions: M2P-HCC liquid biopsy demonstrated good performance for early liver cancer screening in high-risk populations of Hepatitis B-related liver cancer, exhibiting better sensitivity than the combination of AFP and US.

## 1. Introduction

Hepatocellular carcinoma (HCC) is one of the most common malignant tumors worldwide, with low rates of early diagnosis and surgical resection. In China, HCC ranks third in cancer-related mortality [1], and approximately 86% of hepatocellular carcinoma are associated with chronic Hepatitis B virus infection [2]. Currently, the early detection of HCC mainly relies on imaging examinations, serum alpha-fetoprotein (AFP), and proteins induced by vitamin K absence or antagonist-II (PIVKA-II) levels [3]. Nevertheless, the above screening methods have limitations in diagnostic accuracy and sensitivity. Tissue biopsy is widely acknowledged as the gold standard for diagnosing liver cancer, but it remains an invasive procedure not easily accepted by patients, thereby imposing certain clinical limitations. Therefore, there is a need for a more sensitive, specific, and patient-friendly detection method for the early screening and diagnosis of HCC. In recent years, significant inroads have been achieved in molecular biology and genomics, resulting in the advent of liquid biopsy technology, which has greatly facilitated the early diagnosis and treatment of liver cancer [4,5]. A liquid biopsy involves capturing tumor information from the blood, reflecting the body’s systemic tumor information. Compared with traditional needle biopsy, liquid biopsy has the advantages of non-invasiveness, repeated sampling, real-time “update feedback” of tumor burdens, overcoming tumor heterogeneity, and guiding personalized medicine [6]. The clinical application of liquid biopsy in the clinical diagnosis and treatment of tumors includes the analysis of circulating tumor DNA (ctDNA), circulating tumor cells (CTC), circulating microribonucleic acid (miRNA), and exosomes [3,7,8]. Circulating tumor DNA refers to specific mutated DNA fragments released into the peripheral blood by tumors, which can reflect the genomic information of tumors [9,10]. The tumor-specific variations in ctDNA mainly include point mutation, insertion or deletion mutation, abnormal copy number mutation, and methylation level mutation, which can be harnessed for early screening and monitoring liver cancer recurrence [11]. From the perspective of the molecular pathogenesis of liver cancer, methylation changes, and gene mutations play an important role. Various factors, including Hepatitis B virus (HBV), Hepatitis C virus (HCV), alcohol, and non-alcohol-related factors, can interact with the molecular environment in the liver to produce a series of changes, encompassing gene rearrangement, somatic mutation, chromosome rearrangement, copy number variation, and epigenetic changes such as DNA methylation, growth factor pathway changes, etc. [12]. The accumulation of multiple genetic events eventually leads to malignant progression and metastasis of the tumor. It is widely acknowledged that DNA methylation is the most common epigenetic variation. DNA methylation changes can enhance the transcription of proto-oncogenes, silence the expression of oncosuppressor genes, and inactivate DNA repair genes. Methylation changes occur early in the process of liver cancer and precede driver gene mutations. Methylation exhibits tissue specificity, a high detection abundance, and is not influenced by age [13]. Gene mutations involve changing the DNA sequence and affect gene expression and protein synthesis regulation. Gene mutations contribute to telomerase activation to maintain the infinite proliferation of tumor cells and the stability of chromosomes, restricting tumor suppressor gene expression, and thus affecting cell cycle regulation. Moreover, proto-oncogene mutations activate liver oncogenic signaling pathways. In this respect, it has been reported that in patients with Hepatitis B, repeated integration of HBV into the host genome promotes hepatocellular carcinoma [13,14]. In March 2019, the HCCscreenTM Investigational (HIT) study published in the Proceedings of the National Academy of Sciences (PNAS) proposed a new multiomics combined liquid biopsy method, HCCscreen, based on ctDNA mutation, HBV virus integration, and protein markers (including AFP and PIVKA-II). A prospective cohort study of 3793 asymptomatic HBV carriers showed that HCCscreeen improved the detection rate of HCC compared with routine US and AFP screening [15,16]. In a validation cohort of 331 patients, the sensitivity and specificity of HCCscreen for early HCC screening reached 100% and 94%, respectively [15,16]. Subsequently, based on the further optimization and improvement of HCCscreen, a new multi-omics liquid biopsy technique (M2P-HCC) that encompasses the indicators of HCC-related target gene methylation, somatic cell mutation, and protein markers emerged and was included in the “Expert consensus on the role of hematological markers in the early clinical screening of hepatocellular carcinoma” [17]. However, M2P-HCC has been predominantly applied to low-risk HBsAg-positive populations. This study aims to evaluate the effectiveness of M2P-HCC in the screening and diagnosis of Hepatitis B-related liver cancer in high-risk populations.

## 2. Materials and Methods

### 2.1. Patient Population

In this study, patients undergoing long-term follow-ups at the Infectious Diseases Center of West China Hospital of Sichuan University from December 2020 to January 2023 were selected as the study population.

The inclusion criteria of patients were as follows: (1) Age ≥ 18 years and <80 years; (2) Belonging to high-risk and very high-risk groups for hepatocellular carcinoma as defined in the Guideline for stratified screening and surveillance of primary liver cancer (2020 Edition) [18]. Specifically, only patients with a high or very high risk of liver cancer associated with Hepatitis B virus infection were included, while patients with liver cancer caused by other factors were excluded; (3) Patients who provided informed consent and were willing to undergo regular follow-up reviews. Exclusion criteria: (1) Age < 18 years, or ≥80 years; (2) Previously diagnosed HCC or other patients with malignant tumors; (3) People infected with human immunodeficiency virus; (4) Presence of autoimmune diseases (e.g., autoimmune Hepatitis, systemic lupus erythematosus, etc.); (5) Patients with a history of blood transfusion within the last 3 months.

### 2.2. Data Collection

Baseline data of enrolled patients were collected according to the above inclusion and exclusion criteria: (1) General information of patients: name, gender, and age. (2) Medical history collection: family history (mainly family history of liver cancer, Hepatitis B, and other infectious diseases), antiviral therapy drugs, current clinical diagnosis, complicated diseases, etc. (3) Auxiliary examination: Levels of AFP, PIVKA-II, Alanine aminotransferase (ALT), Aspartate aminotransferase (AST), Albumin (ALB), Red cell count (RBC), White blood cell count (WBC), Platelet (PLT), HBsAg full quantification, HBV-DNA quantification, and liver imaging examination results. The above auxiliary examinations were conducted and reported by the clinical Laboratory, Ultrasound Department, and Radiology Department of West China Hospital of Sichuan University.

### 2.3. M2P-HCC Detection

The M2P-HCC detection collection involved the following steps: (1) Peripheral blood collection: 10 mL of fresh EDTA anticoagulant peripheral blood was collected from patients, stored at 10–30 °C, and sent to the laboratory of Chongqing Panson Biotechnology Co., LTD for M2P-HCC detection. The plasma separation process consisted of (a) the sampling vessels were pre-frozen and then mixed upside down 10 times. Afterward, they were centrifuged at 2000× *g* for 10 min at 4 °C. The resulting upper layer of pale-yellow plasma was carefully transferred to a 1.5 mL centrifuge tube, which was then subjected to further centrifugation at 16,000× *g* for 10 min at 4 °C. (b) The supernatant was combined and transferred to a 15 mL centrifuge tube as a plasma sample. (2) Cell-free DNA (cfDNA)extraction: cfDNA was extracted from plasma using the MagMAX™ Cell-Free DNA Isolation Kit (Thermo Fisher Scientific, Waltham, MA, USA), and its concentration was determined by the Qubit dsDNA Hs Assay kit (Thermo Fisher Scientific, Waltham, MA, USA). The size of cfDNA fragments was analyzed by Agilent 2200 TapeStation (Agilent Technologies, Santa Clara, CA, USA) automatic electrophoresis system. A cfDNA sample was considered qualified if the total extraction mass of cfDNA was greater than 5 ng and there was no contamination of long genomic DNA fragments. (3) Rapid amplification of cDNA ends (RACE) library construction: Race-Seq, an endonuclease-dependent amplification sequencing method, was used for library construction. This method can detect both gene mutations (including point mutations, viral integration sites, and other mutation forms) and methylation changes. (4) Determination of protein markers: the concentrations of AFP and PIVKA-I were detected by the chemical luminescence method using the Abbott ARCHITECT i1000SR instrument (Abbott Laboratories, IL, USA). (5) Data model analysis and judgment: The original sequencing data of the RACE-Seq library underwent joint removal and quality control. A genome-wide comparison was conducted, followed by mutation and methylation analysis. Mutation frequency and mutation score were calculated based on liver cancer hotspot mutation and HBV integration information. Similarly, for methylation analysis, the targeted sites contained in the methylation panel were integrated, the methylation state of GC sites in the targeted region was analyzed and calculated to obtain the methylation frequency, and the methylation score was obtained through model training for the training group. Finally, by combining multiple omics markers such as cfDNA methylation score, mutation score, and blood protein markers, and cross-validation relying on machine learning algorithm and medical big data research, the mathematical model of liver cancer screening M2P-HCC was established. This model assigned a liver cancer risk score of 1 to 157 to each sample. The score ranges of 1–86, 86–97, and 97–157 corresponded to negative, warning, and positive results, respectively. A positive result indicates that the subject is highly likely to have liver cancer; a warning result indicates that the subject is likely to have liver cancer or precancerous lesions; a negative result indicates that the subject is unlikely to have liver cancer.

### 2.4. Follow-Up

All patients were observed for 6 to 8 months from the date of blood collection and were regularly followed up in the Infectious Diseases Center of West China Hospital of Sichuan University. AFP, PIVKA-II, and liver imaging were reviewed every 3 months. For patients who exhibited signs suggestive of HCC, further diagnostic tests such as liver ultrasound enhancement, CT enhancement, and tumor-specific MRI were conducted within 1 month. During the follow-up period, patients who were clinically or pathologically diagnosed with HCC or died of liver cancer reached the end of the follow-up period. Patients who did not meet the clinical criteria for HCC were followed up regularly for a minimum duration of 8 months.

### 2.5. Statistical Analysis

All statistical data analyses were performed using STATA version 16.0 (Stata Corp., College Station, TX, USA). The Kolmogorov–Smirnov test was performed to determine whether the data exhibited a normal distribution. Quantitative variables were expressed as the median (centile 25; centile 75). Categorical variables were expressed as numbers (%). Quantitative variables were compared using the Kruskal–Wallis H test. Spearman’s test was applied to determine the relationship between the M2P-HCC score and quantitative clinical data. The diagnostic value of the M2P-HCC score and AFP + US in the diagnosis of HCC patients was assessed by the area under the receiver-operating characteristic curve (AUC). At the same time, a model based on binary logistic regression was established to evaluate the value of the combined diagnosis of M2P-HCC score and AFP + US. From the receiver-operating characteristic (ROC) curve coordinates, the optimal cutoff point associated with the maximum Youden index was determined. Sensitivity, specificity, Youden index, and the cutoff value were used to assess the diagnostic accuracy. Multiple logistic regression analysis was used to determine independent risk factors for liver cancer. All statistical analyses were two-sided; a *p*-value < 0.05 was statistically significant.

## 3. Results

### 3.1. Clinical Characteristics

**Figure 1:**
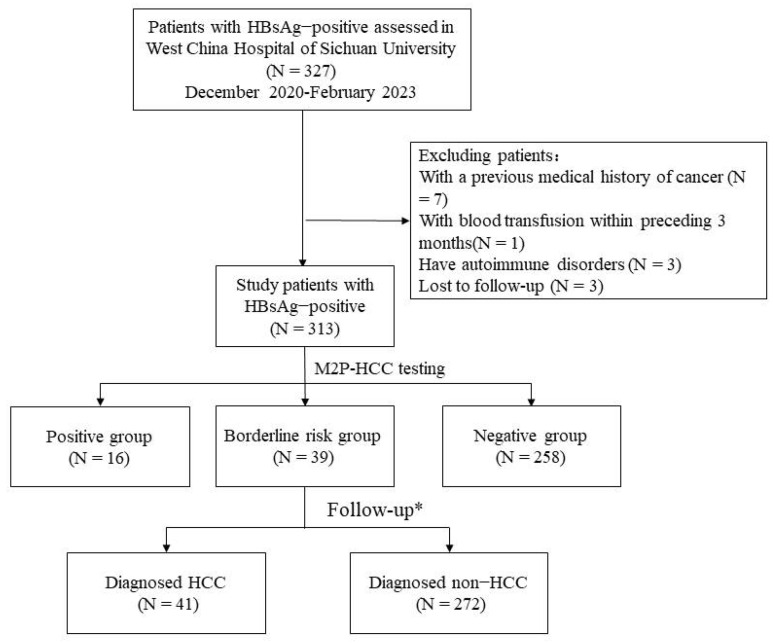
Sample and data-processing workflow. * Follow-up was 6–8 months. Abbreviations: HCC, hepatocellular carcinoma; M2P, methylation, mutation, and protein; HBsAg, Hepatitis B surface antigen.

The selection process for the enrolled subjects is shown in Figure 1. In this study, 327 patients who visited the Infectious Disease Center of West China Hospital of Sichuan University from December 2020 to February 2023 were initially screened according to the inclusion criteria. In total, 14 patients were excluded for the following reasons: (1) History of previous tumors (n = 7); (2) History of blood transfusion within the past 3 months (n = 1); (3) Presence of co-existing systemic immune diseases (n = 3); (4) Cases that did not undergo regular follow-up (n = 3). Ultimately, a total of 313 patients were included.

According to the M2P-HCC score, patients were divided into three groups: positive (97 or higher) (N = 16), borderline risk (86 to 96) (N = 39), and negative (85 or lower) (N = 258) groups. Baseline Characteristics of the three groups are shown in Table 1. According to the follow-up, the patients were divided into the HCC group (N = 41) and the non-HCC group (N = 272). The baseline characteristics of patients with and without HCC at the end of the follow-up are shown in Table 2.

### 3.2. Diagnostic Value of M2P-HCC in Patients at High Risk for Hepatitis B-Associated Liver Cancer

**Figure 2:**
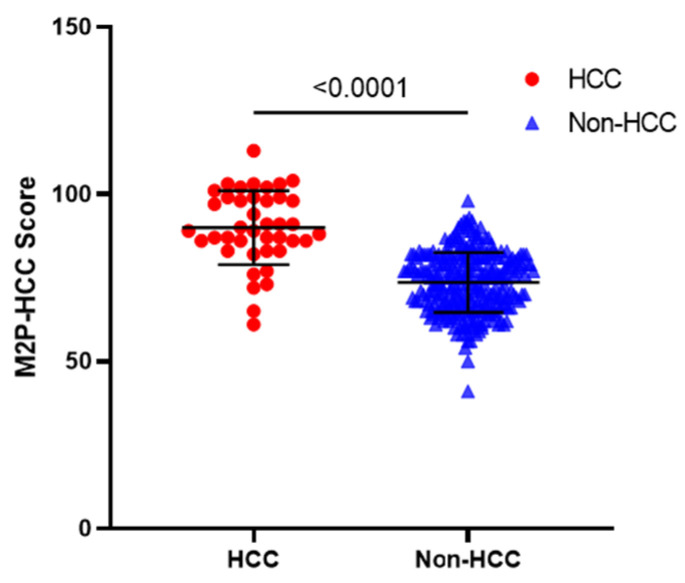
M2P-HCC score in patients with HBV-associated HCC, and non-HCC. Horizontal lines indicate means and standard deviation (solid). The mean M2P-HCC score was 90.0 ± 11.1 in the HCC group and 73.6 ± 9.0 in the non-HCC group (*p* < 0.001). Abbreviations: M2P, methylation, mutation and protein; HCC, hepatocellular carcinoma.

As shown in Figure 2, the M2P-HCC score in the HCC group was significantly higher than in the non-HCC group (*p* < 0.001). The mean M2P-HCC score was 90.0 ± 11.1 in the HCC group and 73.6 ± 9.0 in the non-HCC group, suggesting that the M2P-HCC may have significant value in screening patients at risk of HCC.

**Figure 3:**
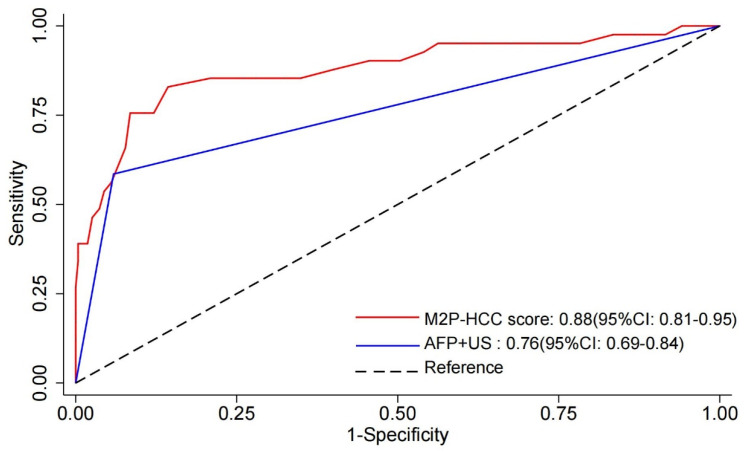
Receiver–operating characteristic (ROC) curve of M2P-HCC score, AFP + US, and the combination of both tests (M2P-HCC score and AFP + US). Abbreviations: M2P, methylation, mutation and protein; HCC, hepatocellular carcinoma; AFP, alpha-fetoprotein; US, ultrasound.

Receiver-operating characteristic (ROC) curve analysis was used to detect the diagnostic value of the M2P-HCC and AFP + US. As shown in Figure 3, the M2P-HCC (AUC = 0.88, 95% CI: 0.81–0.95) exhibited significantly better performance than AFP + US (AUC = 0.76, 95% CI: 0.69–0.84) (*p* = 0.026), with a threshold of 83 for M2P-HCC (sensitivity 82.9% and specificity 85.7%). In contrast, the optimal threshold was 1 for AFP + US (sensitivity 58.5% and specificity 94.1%) (Table 3).

The above results indicated that M2P-HCC liquid biopsy exhibited the superior ability to discriminate HCC patients from non-HCC patients.

### 3.3. Associations between M2P-HCC and Clinicopathological Features in HCC

**Figure 4:**
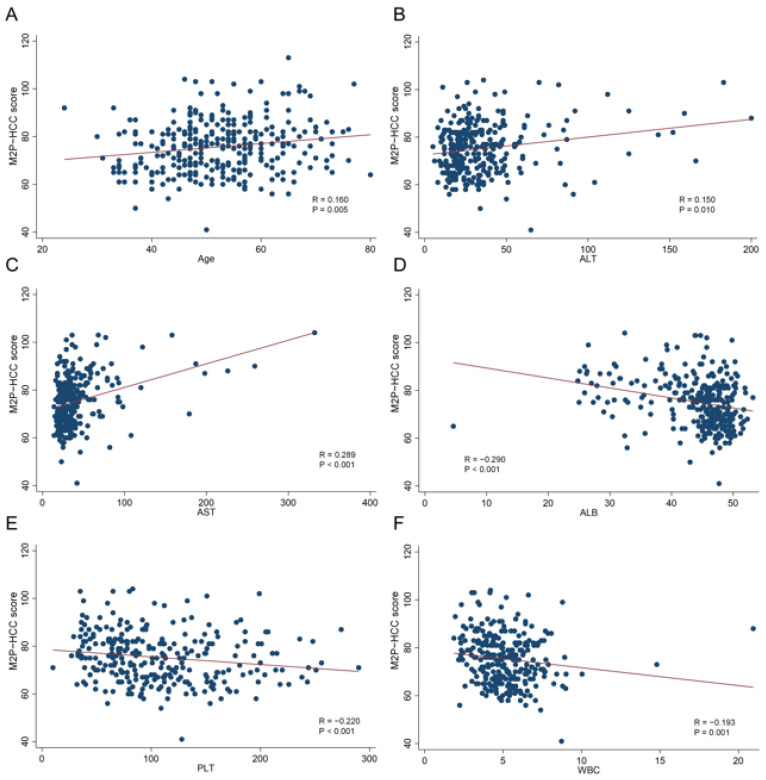
Relationships between the M2P-HCC score and quantitative clinical data in the HCC group. (**A**) Relationship between the M2P-HCC score and age; (**B**) Relationship between the M2P-HCC score and ALT; (**C**) Relationship between the M2P-HCC score and AST; (**D**) Relationship between the M2P-HCC score and ALB; (**E**) Relationship between the M2P-HCC score and PLT; (**F**) Relationship between the M2P-HCC score and WBC.

The M2P-HCC score was positively associated with age (Spearman’s R = 0.160; *p* = 0.005), ALT (Spearman’s R = 0.150; *p* = 0.010), and AST (Spearman’s R = 0.289; *p* < 0.001). The M2P-HCC score was negatively associated with ALB (Spearman’s R = −0.290; *p* < 0.001), PLT (Spearman’s R = −0.220; *p* < 0.001), and WBC (Spearman’s R = −0.193; *p* = 0.001) (Figure 4).

### 3.4. Correlation between HBV Viral Load and the M2P-HCC Score as Well for the AFP + US

**Figure 5:**
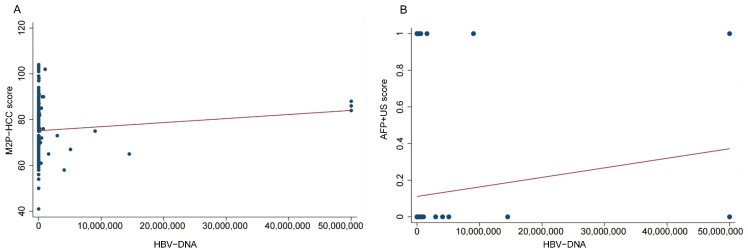
Relationships between the M2P-HCC score, AFP + US score, and HBV-DNA viral load in the HCC group. (**A**) Relationship between the M2P-HCC score and HBV-DNA viral load in the HCC group; (**B**) Relationship between the M2P-HCC score and AFP + US score in the HCC group.

Based on the results presented in Figure 5, it was observed that the PMR value of the M2P-HCC score exhibited a significant positive correlation with the HBV-DNA viral load, as determined by Spearman’s rank correlation coefficient (R = 0.216, *p* < 0.001). Similarly, the PMR value of the AFP + US score demonstrated a significant positive correlation with HBV-DNA viral load (Spearman’s R = 0.419, *p* < 0.001).

Multivariate logistic regression analysis was used to assess the risk factors for HBV-associated HCC. As shown in Table 4, the M2P-HCC Score (OR = 1.16 95% CI 1.09–1.22, *p* < 0.001) was an independent predictor for the occurrence of HCC.

## 4. Discussion

Primary liver cancer, especially HCC, has a high incidence and mortality in China. Despite advancements in diagnosis and treatment technology, the 5-year survival rate of HCC patients remains dismal at 12.1% [19]. In contrast, the 5-year survival rate for early HCC patients with surgical resection is 90% [20]. Indeed, late diagnosis of HCC leads to limited treatment options and shorter median survival times for patients [21]. In the 1980s, Japan implemented a national liver cancer screening program using AFP + US as the primary screening method, and in 1995, AFP-L3 and PIVKA-II were added to the early screening program, marking the screening era of combining triple serological indexes combined with ultrasound. More than 20 years after screening for liver cancer, the five-year survival rate increased from 5.1 percent to more than 40 percent. Meanwhile, the median survival time of liver cancer patients has steadily increased from 4 to 50 months [22]. Therefore, early screening for liver cancer in high-risk groups is crucial.

To our knowledge, this is the first study to apply the M2P-HCC model to a group at high risk of Hepatitis B-related liver cancer. M2P-HCC exhibited better sensitivity and specificity than the conventional AFP + US screening method. However, the association between tumor size and M2P-HCC could not be explored by stratified analysis due to the small number of HCC cases. The current sensitivity and specificity are based on a limited number of HCC cases in this study. It is worth noting that 10 HCC patients did not receive M2P-HCC risk hints. Upon review, DNA methylation changes below the threshold value, along with common gene mutations associated with liver cancer, were detected in these patients. Consequently, these patients were classified as negative in the final model score. However, a small number of cases without HCC were suggested to be at risk of liver cancer by M2P-HCC. These false positive results are not necessarily due to technical errors but could be signals released by dynamic CT/MRI from some small lesions, which may be cleared by the immune system or persist for a long time. Therefore, these results may turn negative at the second screening or continue to suggest liver cancer risk. Increasing the proportion of regular rescreening with M2P-HCC may further increase the positive predictive value of M2P-HCC. Indeed, a high positive predictive value (PPV) and negative predictive value (NPV) are important to reduce unnecessary anxiety and over-examination in non-HCC patients. In addition, since HCC is a disease with an insidious onset and progression, individuals with increased M2P-HCC scores may eventually be diagnosed with HCC over time. These patients should be closely followed up to determine if they are at increased risk for HCC in the future. If confirmed, it would be worth exploring whether more aggressive preventive treatment is necessary for these individuals.

Several shortcomings and limitations in this study should be acknowledged. As mentioned above, the sample size should be expanded to obtain more HCC cases to explore the relationship between tumor size and M2P-HCC. With a larger sample size, stratified analysis results would be more reliable and could help identify the limitations and requirements of M2P-HCC liquid biopsy in the target population for clinical application. Secondly, the follow-up time of 6–8 months was relatively short, and M2P-HCC has not yet been observed to provide early warnings of liver cancer before imaging changes occur. False positive cases identified in this study may develop further into liver cancer in the future, potentially increasing the PPV. Regular follow-up is conducted in all patients, emphasizing M2P-HCC in secondary screenings.

To sum up, this study found that the M2P-HCC multiomics liver cancer risk score model demonstrated good sensitivity for screening in high-risk populations with Hepatitis B-related liver cancer. Moreover, the complementarity between M2P-HCC and AFP + US can reduce the rate of missed diagnosis and misdiagnosis of newborn tumors. Combining the M2P-HCC model with AFP + US can effectively improve the clinical application value of early liver cancer screening, enabling early detection and better treatment opportunities for high-risk groups, ultimately improving prognosis.

## 5. Conclusions

M2P-HCC multiomics liquid biopsy has good sensitivity and specificity for early liver cancer screening in high-risk populations of Hepatitis B-related liver cancer, with better sensitivity than AFP + US.

## Figures and Tables

**Table 1:** Baseline characteristics of the enrolled participants.

Variable	Positive Group (N = 16)	Borderline Risk Group (N = 39)	Negative Group (N = 258)
Age (years)	60.1 ± 9.1	52.2 ± 9.9	52.1 ± 9.9
Male, n (%)	13.0 (81.3)	28.0 (71.8)	189.0 (73.3)
ALT (U/L)	43.0 (26.0–82.0)	32.0 (24.0–48.0)	26.5 (19.0–40.0)
AST (U/L)	66.0 (36.0–122.0)	39.0 (29.0–59.0)	30.0 (23.0–38.0)
AFP (ng/mL)	202.0 (25.1–1155.0)	7.3 (3.0–87.7)	2.9 (2.1–5.3)
PIVKA-II (m AU/mL, %)	121.0 (30.0–176.0)	37.0 (21.0–50.0)	21.5 (18.0–27.0)
ALB (g/L)	43.8 (39.2–45.7)	43.7 (35.8–47.8)	46.2 (43.6–48.2)
PLT (10^9^/L)	83.0 (60.0–133.0)	81.0 (53.0–137.0)	99.0 (68.0–140)
WBC (10^9^/L)	4.2 (3.2–5.2)	4.8 (3.4–5.9)	4.9 (4.0–6.1)
HBV-DNA (+), n (%)			
<1 × 10^2^	8 (50.0)	26 (66.7)	210 (81.4)
1 × 10^2^–1 × 10^4^	4 (25.0)	6 (15.4)	25 (9.7)
>1 × 10^4^	4 (25.0)	7 (17.8)	23 (8.9)
AFP + US (+), n (%)	12 (75.0)	16 (41.0)	12 (4.7)

Abbreviations: ALT, alanine aminotransferase; AST, aspartate aminotransferase; AFP, alpha-fetoprotein; ALB, albumin; PLT, platelet; WBC, white blood cell count; US, ultrasound; PIVKA-II, protein induced by vitamin K absence or antagonist-II; HBV, Hepatitis B virus.

**Table 2:** Baseline characteristics of patients with and without HCC.

Variable	HCC Group (N = 41)	Non-HCC Group (N = 272)	*p*-Value
Age (years)	57.1 ± 9.8	51.9 ± 9.8	0.002
Male, n (%)	29 (70.7)	201.0 (73.9)	0.669
ALT (U/L)	27.0 (21.0–45.0)	28.0 (19.0–41.0)	0.585
AST (U/L)	37.0 (29.0–55.0)	30.0 (23.0–40.0)	0.030
AFP (ng/mL)	43.9 (6.3–185.0)	2.9 (2.1–5.3)	<0.001
PIVKA-II (m AU/mL, %)	37.0 (24.0–121.0)	21.5 (18.0–28.0)	<0.001
ALB (g/L)	43.9 (39.8–45.7)	46.3 (43.3–48.3)	0.001
PLT (10^9^/L)	97.5 (61.0–178.0)	95.0 (67.0–135.0)	0.692
WBC (10^9^/L)	4.6 (3.4–6.3)	4.9 (4.0–6.0)	0.473
HBV-DNA (+), n (%)			0.001
<1 × 10^2^	23 (56.1)	221 (81.3)	
1 × 10^2^–1 × 10^4^	8 (19.5)	27 (9.9)	
>1 × 10^4^	10 (24.4)	24 (8.8)	
AFP + US (+), n (%)	24 (58.5)	16 (5.9)	<0.001

Abbreviations: ALT, alanine aminotransferase; AST, aspartate aminotransferase; AFP, alpha-fetoprotein; ALB, albumin; PLT, platelet; WBC, white blood cell count; US, ultrasound; PIVKA-II, protein induced by vitamin K absence or antagonist-II; HBV, Hepatitis B virus.

**Table 3:** The diagnostic value of M2P-HCC score, AFP + US, and the combination of both tests (M2P-HCC score and AFP + US) in discriminating HBV-associated HCC.

Variable	Sensitivity	Specificity	Youden Index	Cutoff
M2P-HCC	82.9%	85.7%	0.69	83.00
AFP + US	58.5%	94.1%	0.53	1.00

Abbreviations: M2P, methylation, mutation and protein. HCC, hepatocellular carcinoma. AFP, alpha-fetoprotein. US, ultrasound.

**Table 4:** Independent risk factors for the development of HCC.

Variable	OR	95% CI	*p*-Value
Age (years)	1.05	1.00–1.09	0.052
Male, n (%)	0.97	0.36–2.59	0.956
AFP + US	8.04	2.89–22.41	<0.001
M2P-HCC Score	1.16	1.09–1.22	<0.001

Abbreviations: M2P, methylation, mutation and protein; HCC, hepatocellular carcinoma; AFP, alpha-fetoprotein; US, ultrasound; CI, confidence interval; OR, odds ratio.

## Data Availability

The data that support the findings of this study are available from the corresponding author, Xuezhong Lei, upon reasonable request.
